# Household disaster management capacities in disaster prone II area of Mt. Slamet

**DOI:** 10.4102/jamba.v11i1.649

**Published:** 2019-07-04

**Authors:** Diah S. Dewanti, Dusadee Ayuwat, Sekson Yongvanit

**Affiliations:** 1 Department of Development Science, Khon Kaen University, Khon Kaen, Thailand; 2 Department of Economics Development, Universitas Muhammadiyah Yogyakarta, Bantul, Indonesia; 3 Department of Sociology and Anthropology, Khon Kaen University, Khon Kaen, Thailand

**Keywords:** disaster management, Mt. Slamet, Indonesia, disaster prone II, parth analysis

## Abstract

Disaster prone II in Mt. Slamet, Indonesia presents the highest risk for human settlement. To live in this natural disaster-prone area, specific household characteristics are essential. Household capitals and transformation in process and structure were supported by the disaster management framework. However, households in disaster prone II area had limited assets and were required to identify factors influencing disaster management. To study the factors influencing household disaster management capacities, this research, using the sample measurement of Becker and Hursh-Cesar, collected data of 538 households spread across five villages in the disaster prone II area of Mt. Slamet. Sequential mixed methodology combining both qualitative and quantitative research methods were used: samples in the Rukun-Warga-level area were collected by a two-stage stratified random sampling, and to choose the sample of households systematic random sampling was employed. Path analysis through Stata was carried out to analyse the direct and indirect factors supporting disaster management capacity, and multicollinearity was tested before path analysis. This research found direct and indirect effects of household characteristics and household capitals on disaster management. This could be influenced by the transformation in process and the structure of the local government. The quantitative result has been confirmed by the result of the qualitative methodology. Social capital owned by households in disaster-prone area supports disaster management practices. The household relationship and networking access has been strongly supported by disaster management capacities. Disaster management capacities of households in disaster prone II areas could be improved by both internal and external factors. Internal factors include supporting the household members’ health and increasing the size of land and vehicle owning. Meanwhile, external factors has been applied by the policy published by government as to improve the social and cultural belief of households.

## Introduction

It is essential for households in disaster-prone areas to have disaster management capacities to ensure their safety and survival when faced with hazardous events (Sutton & Tierney 2006). To survive environmental hazards, a four-option framework described by Lewis (1999) and Wisner et al. (2004) could be applied. However, the fourth framework element, ‘Live with hazard and risk’, is appropriate for households that lived in the area with the highest risk of hazard. This framework is integrated into the ‘environmental threats and opportunities’and accepts disasters as a part of life and protects their livelihoods. Cannon, Twigg and Rowell (2003) and Twigg (2004) used the sustainable livelihoods approach (SLA) in their previous study to reduce disaster risk. Sustainable livelihoods approach by Department for International Development (DFID) explores with a *pentagon* of human capital, social capital, natural capital, physical capital and financial capital, which is influenced by outside policies, institutions and processes of living in a disaster area. Household is the proper unit analysis applied in each of the sustainability livelihood approaches. Based on the literature review, the concept could be applied to support vulnerable households in areas prone to high risk of disasters.

Indonesia is located in the ring of fire, which is vulnerable to natural hazards, one of which is volcanic disaster. This leaves Indonesia very vulnerable to the impact of natural hazards. More than 150 active stratovolcano types are spread around all of the big islands, one of them being Mt. Slamet. It is the second highest mountain (3428 m) in Java Island, with more than 50 eruptions recorded since 1988. Mt. Slamet borders five districts in Central Java province, namely, Brebes, Banyumas, Purbalingga, Tegal and Pemalang. From March 2014 to January 2015, Mt. Slamet started to have unpredictable fluctuation in seismic activity. Huge explosions occurred in March, August and September 2014, which were the biggest explosions compared to the previous ones in the 20th century. The Indonesian government has identified three disaster-prone areas based on the length of the mountain peak (Regulation of Minister of Energy and Mineral Resources of Republic Indonesia 2011). Disaster prone III, located 0 km–4 km from the peak, is the highest risk level, and this area is forbidden for human settlement or any other activity.

Dewanti and Ayuwat (2015) described the condition of disaster-prone volcanic areas, one of them being Sawangan village, located on the border of disaster prone II and III. This study highlighted the limitations on capital and indicated that they were living a self-sufficient life, with little support from the government. Hence, minimal capital was owned by the households in the disaster prone II area, as they had already carried out volcanic disaster management. Several areas in the disaster prone II area of Mt. Slamet had already forged their survival practices based on indigenous knowledge. The other area, Guci village, is located in the disaster prone II area of Mt. Slamet. Sawangan hamlet and Guci village were both partly located in the disaster prone II area of Mt. Slamet and practised volcanic disaster management differently. Sawangan implemented disaster management supported by social kinship, while Guci applied a top-to-bottom leadership system driven by local government construction. It created different disaster management capacities to be employed by the households (Dewanti, Ayuwat & Yongvanit 2016). However, the outside institutions, policies and processes of living in the disaster prone II area influenced the household capitals as household assets to be applied in a disaster management capacity.

However, an analysis was required to identify which household capital could influence the disaster management capacities of the households that lived in the disaster prone II area. Households that lived in the disaster prone II area were mostly engaged in farming and tourism sectors. Some households had urban living standards, while others still had underdeveloped livelihoods. Nevertheless, this article is contributed to fulfil the disaster management influenced by the capitals of households that lived in the disaster prone II area of Mt. Slamet, Indonesia.

This research focussed on the concept of disaster management capacities for households. The concept of livelihood and pointed household as the concept of agency that applied the disaster management were described as independent variables (IVs). Disaster is defined as a serious disturbance to the functioning of a society that influences loss of human life, materials and environment, which impacts the capability of the society to cope using its own resources (UN 1992). To analyse factors influencing disaster management, we have to start by comprehending the meaning of disaster and disaster management based on local perspectives.

**FIGURE 1:**
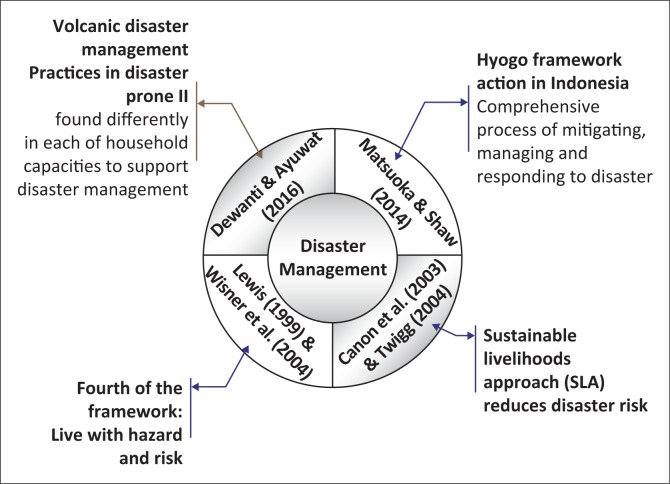
Theoretical framework based on meaning and perspective of disaster management.

The development of disaster management cognition is described in Figure 1, with the meaning of disaster management and its perspective. Lewis (1999) and Wisner et al. (2004) described how to live with hazards and risk in a disaster-prone area. High-risk areas need to balance their resilience capability with their own resources through SLA for mitigating disaster risk (Cannon et al. 2003; Twigg 2014). Hyogo Framework Action (HFA) was established in Indonesia as a comprehensive process for mitigating, managing and responding to disaster. It is in line with the international disaster management framework, which was developed using local wisdom in each area (Matsuoka & Shaw 2014).

Hyogo Framework Action was formulated in 2005–2015, which focussed on reducing the risk of vulnerable population from many disaster scenarios. The framework explained the cycle of the disaster event before, during and after the occurrence of the disaster as stated in the mitigation, preparedness, response and recovery phase. Disaster management is defined as comprehensive and integrated activities to cope with the cycle of disaster events. This research is aimed at analysing the disaster management capacities that are linked to the integrated activities on mitigation, preparedness, response and recovery phase. However, there are factors that influence the disaster management, such as household characteristics, household capitals and transformation on process and structures.

Based on literature review and development of concept, this research define the definition of term for disaster management and livelihoods as follow:

- disaster management as a household plan, practice and action for those who live on the border of the disaster prone II area to reduce the risk and recover from the impact of volcanic disaster through integrated activities on mitigation, preparedness, response and the recovery phase
- livelihoods as household capitals that influence the transforming structure and process to have access to livelihood resources.

Transforming structure and process as an institution and policy interaction supported by the local culture and belief practices. The definition of terms brought three hypotheses that need to be examined in this study:

- **Hypothesis 1:** Households that lived in disaster prone II areas having more specific characteristics on occupation, dependency ratio, labour force, healthy house members, migration, size of land, amount of machine goods and vehicles influenced disaster management.
- **Hypothesis 2:** Household capitals of those who lived in disaster prone II areas, which consist of certain human capital, social capital, natural capital, physical capital and financial capital, influenced disaster management.
- **Hypothesis 3:** Transformation on process and structure, which consists of land-use management, spiritual practices and cultural implementation for households that lived in the disaster prone II areas, influenced disaster management.

Through the hypotheses, a semi-structured guideline was developed and factors influencing disaster management practices were described. Qualitative approach was used to further describe how each of the factors in SLA supports disaster management practices. This research aimed to describe the factors influencing the livelihoods of the households that lived on the border of the disaster prone II area of Mt. Slamet, Indonesia.

## Research methods and design

This study used sequential mixed methods, which start with quantitative methods, and had the household as the unit of analysis. As the study area was in the disaster prone II area, the population was spread across five subdistricts of 4268 households. To select the study area, a two-stage stratified sampling was used to choose three districts based on the most impacted areas during previous eruptions. Villages in each district were chosen based on their location in the disaster prone II area of Mt. Slamet. The samples were measured by Backstrom and Hursh-Cesar (1981) formula by increasing the suggested sizes based on calculation of the sample size through measurement of confidence level, sampling error, population heterogeneity and population size. The calculation was as follows: multiply the population with 95% confidence level and ±5% sampling error of 50/50 split. A sample size of 357 households was derived and to enlarge the size of the sample, using Backstrom and Hursh-Cesar’s formula, it was multiplied by 1.5; thus, a total sample size of 538 households was derived. Systematic sampling was used to choose the sample households and thereby the respondents. The systematic sampling was measured with interval 9th and chosen from a list of households in each village (see Table 1). This study identified three dimensions as IVs – (1) household characteristics, (2) capitals of households and (3) transformation on process and structure – and disaster management as the dependent variable.

**TABLE 1:** Distribution of sampling of households that lived in disaster prone II area.

Sub-district	Village	Population	Percentage	Sample size
Bumijawa	Sawangan	664	14.35	78
	Guci	1020	22.04	118
Bojong	Dukuh Tengah	830	17.95	97
Baturaden	Ketenger	1012	21.87	117
Pulosari	Gunungsari	1102	23.81	128
**Total**		**4268**	**100.00**	**538**

*Source:* Local village documentation, 2017

Household characteristics consisted of occupation, dependency ratio, labour force, number of healthy household members, migration, amount of electrical goods and vehicles, and size of farming land. All of the indicators used ratio data scale except for occupation, which used the ordinal scale. However, there were two dummy variables, namely, farming and non-farming occupation. Capitals of households consisted of human capital, social capital, natural capital, physical capital and financial capital. All of the variables used ratio and interval data scales. Transformation, process and structure, consisting of land-use management, spiritual practices and cultural implementation, used interval data scales. Disaster management, which consisted of mitigation, preparedness, response and recovery phase, used the interval data scale. The researchers developed these indicators from the field study through a qualitative approach and the literature review of previous studies. Previous research has been conducted as a qualitative approach to design the instruments of this study. A quantitative study was conducted by structured interview schedules (SIS) with multivariate analysis using the Stata version 14 programme. Content validity and measurement of reliability were performed, with Cronbach’s alpha at 0.908, to check the quality of the data. Content validity was performed by sending the questionnaire to an expert in environmental studies and disaster management to make improvements to the questionnaire. The experts were selected based on their expertise: two experts from the field of natural resource management and one from statistics. As this study employed multivariate analysis on path analysis, multicollinearity diagnostic was employed to examine the correlation between IVs. This could cause several problems with the estimation of *β* (unstandardised beta coefficient) and interpretation. Multicollinearity could be used in three ways: (1) examination of the correlation matrix, (2) variance inflation factor (VIF) and (3) eigen-system analysis of correlation (Joshi 2012). This study employed VIF measurement to test whether the data had the problem of multicollinearity. Among 25 IVs, no problems of multicollinearity were found, with VIF ranging between 1.09 and 2.15 or less than 10.

The second phase of the study was carried out using qualitative methods. These methods used focus group discussions for three different groups: head of the village, rescue team and head of the household. Each group represented five villages that were selected by previous approach, that is, quantitative methods. It was carried out separately in each group to answer the further description of factors relating to disaster management practices.

### Ethical considerations

Ethical approval to conduct the study was obtained from Khon Kaen University (registration number: HE 583022).

## Discussion

**FIGURE 2:**
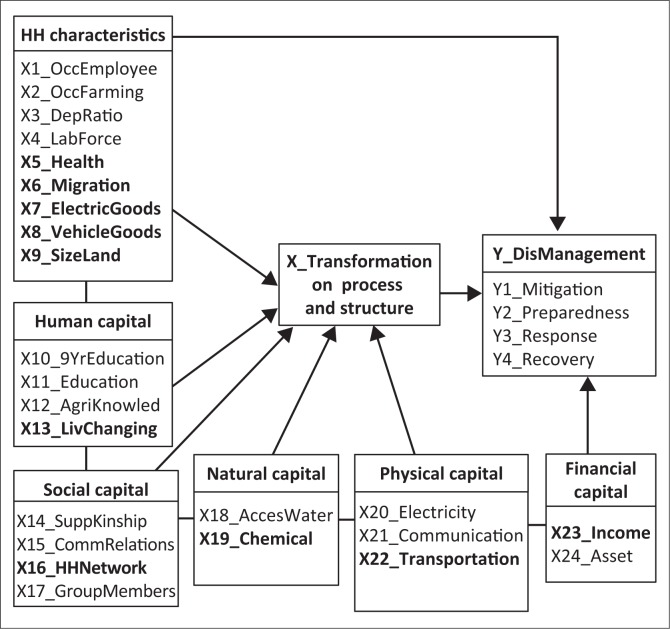
Conceptual framework before path analysis.

The results of this study described the factors influencing disaster management in three dimensions: household characteristics, capitals of households and transformation of process and structure. Before the path analysis, this study used the conceptual framework model as depicted in Figure 2.

The conceptual framework has been arranged by theoretical reviews and field study in the disaster prone II area of Mt. Slamet. Before the path analysis, the variables need to be analysed for correlation between them. This study used a multicollinearity test to measure the correlation matrix and VIF. The aim of the multicollinearity test is to decrease the exact relationship between predictor variables. To identify the goodness of fit of the model, the study used the Pearson chi-square analysis. This analysis indicates the ‘badness of fit’ of the model and whether the outcome frequencies follow a specified distribution. It is applied in both categorical data and unpaired data from large samples. The computational procedures comprised five steps: (1) calculating the chi-squared test statistics (*χ*²), (2) determining the degrees of freedom of the statistics, (3) considering the desired level of confidence of the result of the test, (4) comparing χ² to the critical value from the chi-squared distribution with *df* (degree of freedom) and adjusted confidence level and (5) determining whether the null hypothesis is accepted or rejected (H_0_ = no differences between distribution). Through this identification, the whole of the *χ*² of the 26 variables exceeded the statistical value, which resulted in the rejection of the null hypothesis, or stated that there is a difference between observed data and the expected value.

**FIGURE 3:**
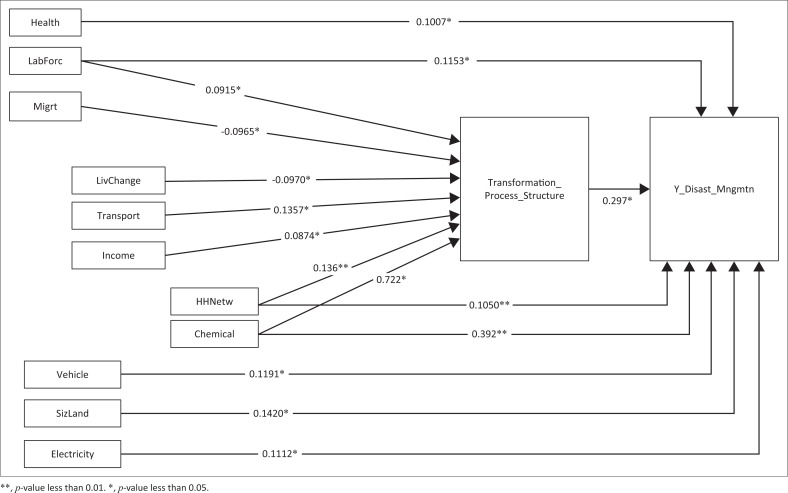
Path analysis result.

This path model has *R* squared as 33.44% and is described in Figure 3. There were eight IVs that had a direct effect (DE) on disaster management: health of household members, labour force, number of vehicles, size of farming land (household characteristics), household networking to others (social capital), chemical fertiliser (natural capital), access to electricity (physical capital) and transformation of process and structure. Chemical fertiliser utilisation and household networking to others were found to be significant at 0.000 with the unstandardised coefficients as 0.392 and 0.105, respectively.

There were seven IVs having indirect effect (IE) on disaster management through transformation of process and structure as the mediator: labour force, migration (household characteristics), livelihood changing (human capital), household networking to others (social capital), chemical fertiliser (natural capital), access to transportation or road (physical capital) and income (financial capital). Migration and livelihood changing were found to be unstandardised coefficients with values of -0.0965* and -0.0970*, respectively (with the *p*-level of significance at 0.05). It was further found from the data that most household members who migrate were men, the heads of the household and/or married sons, men who had moved out for education or those who were working in another province. Ho et al. (2008) and Kung and Chen (2012) stated that the women were better prepared for disaster management; however, during an eruption, they were found wanting with regard to evacuating their family to a safer place. Furthermore, labour force, household networking to others and chemical fertiliser utilisation had both DE and IE on disaster management.

Based on quantitative results, the focus group discussions were started with the heads of the villages. They explained their practices to reduce disasters based on ancestral practices, for example, sacrificing of a goat head to Mt. Slamet. Therefore, households refused to move out from the disaster prone II area and continued their livelihoods of farming and tourism entrepreneurship in such high-risk areas. However, the eruption in 2014 created greater awareness on how gravely Mt. Slamet could harm the households during an eruption. As a participant stated:

… the ancestor has already stated to all household in here. Mt. Slamet will explode as the same time as end of the world. We do not need to be scared. But, on 2014, suddenly Mt. Slamet expended very big explosion sound with earthquake; now we consider to be aware of Mt. Slamet …. (Participant 4, male, farmer, 54 years)

Local government appointed by the villagers took the lead role in introducing disaster maps. Therefore, several areas are aware of both the likely dangers in their place of habitation and the evacuation area or meeting point during an eruption. On the contrary, one of the key informants from Guci explained that the evacuation signs were missing because of the vested interests of another faction in the government who fear that revenue from tourism will decrease. As stated by the participant:

… everytime we saw from TNI ABRI (Indonesian soldier) put evacuation sign and meeting point sign; in the next day it will automatically be missing. Someone takes it out and we do not know who and why they take it. You can see Guci does not have the signs now…. (Participant 1, male, farmer, 46 years)

When Mt. Slamet indicates higher seismic activities, the preparedness phase begins. During this phase, the households need to be aware of and prepare the survival kit as the next phase could occur anytime. To recognise the warning alarm or warning system owned by each local area is the most important capacity of each household member. Networking with other households living in non-risk areas is needed to encourage households that live in disaster prone II areas. Improving the physical capital, such as road access, plays an important role in the preparedness phase before the actual eruption or response to an eruption. However, one of the places closest to the peak of Mt. Slamet has a limitation with the quality of infrastructure, mainly concerning the road for evacuation. Statement by participant:

… local government has agreed but the making of the road took long time. We are running out of our patience, so we keep it that way. The most important road is the evacuation road; it is the road that made us die during evacuation because we could not pass using car or motorcycle, but only bare foot. How can we do the evacuation during Mt. Slamet eruption …. (Participant 3, male, farmer, 62 years)

Limitations with regard to physical capital resulted in less disaster management practices. Transformation in process and structure is addressed related to land-use management, spiritual implementation and cultural practices. Land-use management is the practice of cutting lesser number of trees and retaining the trees to strike an environmental balance with nature. In this area, most of the households were Muslim and followed the practices of Islam. Cultural practices have been described as the traditional and customary practices of a cultural group, for example, the Ruwat Bumi event. Households from Guci described Ruwat Bumi as an annual event that is celebrated in collaboration with the local government for Mt. Slamet, and most of the households invite tourists to their area and thus increase their income.

## Conclusion

From the findings of the DE and IE among 12 IVs, it could be concluded that variables that have DE and IE were supporting disaster management practices for households. Those were the labour force and utilisation of chemical fertiliser. Both these variables positively affected disaster management. The number of households that have members of working age had a total effect of 0.2775, with a *p*-value of 0.05 (see Table 2). Utilisation of chemical fertiliser had a total effect of 1.411, with a *p*-value of 0.01. Based on the measurement of path analysis, it could be described that when households had members in the working age, it could raise the disaster management capacity to 1.441. Furthermore, when household members utilize non-chemical fertilizer in their fields, as much as 1 meter, it could improve disaster management capacity to 0.7912.

**TABLE 2:** Total effect of factors influencing disaster management and the qualitative findings.

Independent variables	Direct effect	Indirect effect	Total effect	Qualitative findings
Labour force	0.1153	0.9150 × 0.1773 = 0.1622	0.2775	Providing training to household members who are in the age of labour force group
Number of healthy members in household	0.1007	-	0.1007	Households need to be trained as part of a rescue team
Migration	0.0000	(0.0965) × 0.1773 = (0.0171)	(0.0171)	Recognise the vulnerable group in which the household members have mostly migrated to other areas
Amount of vehicles	0.1191	-	0.1191	Improve the road access
Size of farming land	0.1420	-	0.1420	Increase the green environment awareness
Livelihoods changing	0.0000	(0.0970) × 0.1773 = 0.0172	(0.0172)	Less changing of working characteristics could support their process of earning an income and disaster management capacities
Household networking to others	0.1050	0.136 × 0.1773 = 0.0241	0.1291	Prepare to have evacuation networking on response phase
Chemical fertiliser utilisation	0.7220	0.392 × 0.1773 = 0.0695	0.7912	Participate on green environment awareness
Access to electricity	0.1112		0.1112	Updated information among others by media
Income	0.0000	0.0874 × 0.1773 = 0.0155	0.0155	Income plays an important role during the eruption and recovery process
Transformation of process and structure	0.2970	-	0.2970	Land-use management, spiritual belief and cultural practices support the disaster management practices to support their living in high-risk areas

An interesting finding was that migration had a DE with an unstandardised beta coefficient of 0.0171, and a *p*-value of 0.05; it could be described that households that had more than one member who migrated could have a decrease of 0.0171 points in disaster management practices. Koks et al. (2015) explained migration as the impact in social vulnerability from In this study, migration of household members had a negative impact on the disaster management capacity of the household itself and increased the social vulnerability. The findings were drawn from the factors supporting improved disaster management based on household capacity. Furthermore, it could predict which parts could be improved in the household disaster management guideline to strengthen households that live at the high-risk border of disaster prone II area of Mt. Slamet, Indonesia.

Based on the findings of this study, it can be concluded that the first hypothesis found that five IVs had DE and/or IE on disaster management: (1) labour force, (2) number of healthy household members, (3) migration, (4) amount of vehicles and (5) size of land. These were the specific household characteristics which could influence the disaster management for households that lived in the disaster prone II area of Mt. Slamet. The result of this hypothesis is in line with the concept of Onuma, Shin and Managi (2017), who described that disasters could relatively be handled by several characteristics, for example, age: a younger head of a household is more likely to be better prepared for disaster. It also includes the number of healthy household members who could be described as a non-vulnerable group concerning disaster management assessment. As households that lived in the disaster prone II area mostly relied on natural resources and lived in rural households, the size of land influenced disaster management (Card 1999).

The second hypothesis is in line with the core concept of the research by Lewis (1999) and Wisner et al. (2004), who stated that volcanic disasters could be dealt with by using four frameworks. However, in this study, the researchers used the fourth framework, which was described as ‘live with hazards and risk’, using household assets or capitals to cope or deal with volcanic disaster. Hoffmann and Muttarak (2017) mention one of the disaster phases, preparedness, through social capital and disaster risk perception of the household. It strengthens the capitals of households through the disaster management capacities in each of the phase. In summary, the capitals of households influenced the disaster management directly, indirectly or both.

The third hypothesis described three parts that were explained as land-use management, spiritual implementation and cultural practices. However, the variable of transformation of process and structure had a positive influence on disaster management and played a role to bridge the gap between some variables of household characteristics and the capitals of households. In line with this hypothesis, the researcher has proven that the transformation of process and structure has a positive influence on disaster management as the outcome activities for households that lived in the disaster prone II area of Mt. Slamet. It is supported by DFID (2000) and Scoones (1998) who described transformation of process and structure as the engine of households to achieve an outcome. In this study, spiritual and cultural practices influenced the disaster management capacities of the households. The cultural practice recognised as Ruwat Bumi is an annually held collaboration between households that lived in the disaster prone II area and the local government. Cultural practices are the engine of household beliefs for disaster management capacity. As it is their heritage inherited from their ancestors, it is proof of the transformation of process carried out by cultural practices not only by the households but also supported by the structure of the government. As this research is important in that it contributes to fulfil the disaster management concept in disaster prone II area, the research materials related to this article can be accessed for completing the academic research gap.
